# Skeletal Muscle-Derived Stem/Progenitor Cells: A Potential Strategy for the Treatment of Acute Kidney Injury

**DOI:** 10.1155/2016/9618480

**Published:** 2016-03-16

**Authors:** Egle Pavyde, Romaldas Maciulaitis, Mykolas Mauricas, Gintaras Sudzius, Ernesta Ivanauskaite Didziokiene, Arvydas Laurinavicius, Neringa Sutkeviciene, Edgaras Stankevicius, Justinas Maciulaitis, Arvydas Usas

**Affiliations:** ^1^Institute of Physiology and Pharmacology, Medical Academy, Lithuanian University of Health Sciences, LT-44307 Kaunas, Lithuania; ^2^Department of Nephrology, Medical Academy, Lithuanian University of Health Sciences, LT-50009 Kaunas, Lithuania; ^3^State Research Institute Centre for Innovative Medicine, Vilnius University, LT-01102 Vilnius, Lithuania; ^4^National Center of Pathology, Vilnius University Hospital Santariskiu Clinics, LT-08406 Vilnius, Lithuania; ^5^Department of Pathology, Forensic Medicine and Pharmacology, Faculty of Medicine, Vilnius University, LT-03101 Vilnius, Lithuania; ^6^Department of Non-Infectious Diseases, Veterinary Academy, Lithuanian University of Health Sciences, LT-47181 Kaunas, Lithuania; ^7^Institute of Sports, Medical Academy, Lithuanian University of Health Sciences, LT-50275 Kaunas, Lithuania

## Abstract

Skeletal muscle-derived stem/progenitor cells (MDSPCs) have been thoroughly investigated and already used in preclinical studies. However, therapeutic potential of MDSPCs isolated using preplate isolation technique for acute kidney injury (AKI) has not been evaluated. We aimed to characterize rat MDSPCs, compare them with bone marrow mesenchymal stem cells (BM-MSCs), and evaluate the feasibility of MDSPCs therapy for gentamicin-induced AKI in rats. We have isolated and characterized rat MDSPCs and BM-MSCs. Characteristics of rat BM-MSCs and MDSPCs were assessed by population doubling time, flow cytometry, immunofluorescence staining, RT-PCR, and multipotent differentiation capacity. Gentamicin-induced AKI model in rat was used to examine MDSPCs therapeutic effect. Physiological and histological kidney parameters were determined. MDSPCs exhibited similar immunophenotype, stem cell gene expression, and multilineage differentiation capacities as BM-MSCs, but they demonstrated higher proliferation rate. Single intravenous MDSPCs injection accelerated functional and morphological kidney recovery, as reflected by significantly lower serum creatinine levels, renal injury score, higher urinary creatinine, and GFR levels. PKH-26-labeled MDSPCs were identified within renal cortex 1 and 2 weeks after cell administration, indicating MDSPCs capacity to migrate and populate renal tissue. In conclusion, MDSPCs are capable of mediating functional and histological kidney recovery and can be considered as potential strategy for AKI treatment.

## 1. Introduction

Acute kidney injury (AKI) is a serious and frequent clinical condition with a high mortality rate [1]. The incidence of this life-threatening complication is rapidly increasing, especially among critically ill hospitalized patients and among those undergoing major surgical procedures [1–3]. The main treatment options for AKI include supportive care and renal replacement therapy. Despite the application of renal replacement therapy for AKI patients, the mortality rates remain as high as 50–60% [1]. Serious threat to human life shows the importance of the evolving problem and the need for the development of innovative treatment options. During the last several decades, the growing attention is drawn to the possible application of cellular therapies for the treatment of AKI.

Mesenchymal stem cells (MSCs), also known as mesenchymal stromal cells, are undifferentiated, self-renewable, multipotent adult stem cells, which originate from the mesoderm germ layer [4]. MSCs can differentiate into a broad range of different cells, including both mesenchymal and nonmesenchymal lineages, such as bone, cartilage, muscle, fat, neurons, and cardiomyocytes [4–10]. The principal, most widely used source of MSCs is the bone marrow. However, MSCs can also be isolated from various other tissues such as muscle, fat, periosteal tissue, or umbilical cord blood [11–13]. MSCs are widely used due to their advantageous characteristics, including the possibility to use MSCs for both autologous and allogeneic therapies.

MSCs, and particularly bone marrow mesenchymal stem cells (BM-MSCs), are the most frequently used stem cell type for the prevention and treatment of kidney diseases. Various studies have shown the effectiveness of the BM-MSCs therapy for kidney regeneration after gentamicin, cisplatin, and ischemia-reperfusion induced AKI in animal models [14–20]. However, the mechanism of action of BM-MSCs in renal regeneration after AKI still remains controversial and is a matter of debate. Several studies have reported that injected BM-MSCs improve the kidney function and structure directly, by infiltrating the kidney and populating the renal tissue [17–19]. Other studies have found no evidence of stem cell incorporation into the renal tubules and suggested the paracrine effects as the main mechanism of action of BM-MSCs in treating AKI [14–16, 20].

Skeletal muscle-derived stem/progenitor cells (MDSPCs) are mesenchymal stem cell lineage, possible predecessors of muscle satellite cells [21]. MDSPCs are multipotent cells, demonstrating high self-renewal, long-term proliferation capacities and promoting endogenous tissue repair by secreting trophic factors [21–23]. MDSPCs have already been used in preclinical studies and clinical trials to repair, regenerate, and restore a variety of different tissues following acute injury or tissue destructive diseases, such as muscular dystrophies, bone and cartilage injuries, peripheral nerve damage, and urinary bladder dysfunction [24–28]. To date, the therapeutic potential of MDSPCs isolated using preplate isolation technique [12] has not been evaluated for kidney regeneration after AKI. Considering the characteristics of MDSPCs and the origin from the same mesodermal germ layer as the renal tissue, we hypothesized that MDSPCs may become a potential new strategy for the treatment of AKI. In our study, we aimed to characterize rat MDSPCs, compare them with BM-MSCs* in vitro*, and evaluate the feasibility of the MDSPCs therapy for the gentamicin-induced AKI in a pilot study in rats.

## 2. Materials and Methods

### 2.1. Animals

All the study-related procedures were approved by the Animal Health and Welfare Department, State Food and Veterinary Service. Male 2–5-week-old Wistar rats were used for the isolation of BM-MSCs and MDSPCs. Female 8–12-week-old Wistar rats weighing 150 to 250 g were used for the experiments* in vivo*. The rats were housed in metabolic cages under ordinary conditions (24 ± 1°C, 12-hour light and 12-hour dark cycles) and allowed free access to food and water.

### 2.2. Isolation of Bone Marrow Mesenchymal Stem Cells

Rat BM-MSCs were isolated using a modified previously described protocol [29]. Briefly, 4-5-week-old male Wistar rats weighing 100–150 g were euthanized, hind limbs were removed from the trunk of the body, and the remaining muscle and connective tissues were removed. After cleaning, BM-MSCs were harvested under sterile conditions, by cutting the ends of bones below the end of the bone marrow cavity. A complete BM-MSCs proliferation medium (PM) filled syringe with 27-gauge needle was inserted into the medullar canal, a marrow plug was flushed out of the bone cut end, and cell suspension was filtered through a 70 *μ*m cell strainer. BM-MSCs were cultured in T-25 culture dish and incubated for 12 hours without disturbance. Afterwards, the nonadherent cells were removed by changing the PM. The medium was changed every 12 hours for up to 72 hours of initial culture (passage 1). Afterwards, fresh medium was added every 3 days. After 2 weeks, the cells were seeded in a T-75 flask and maintained in the PM with media change every 3 days. New passages were obtained every 5-6 days at 70–80% confluence.

### 2.3. Isolation of Muscle-Derived Stem/Progenitor Cells

Rat MDSPCs were isolated as previously described [12]. Briefly, 2-3-week-old male Wistar rats weighing 40–50 g were euthanized, and the hind limb skeletal muscles were dissected, placed in a sterile Petri dish, washed 3 times with HBSS, and dissected from other tissues. Afterwards, the muscles were minced into a suspension and washed. Enzymatic digestion was performed by the incubation with 0.2% collagenase type XI solution (Sigma-Aldrich) for 60 min, followed by 2.4 units/mL dispase solution for 45 min and 0.1% Trypsin-EDTA solution for 30 min. A cell pellet was then resuspended in MDSPCs PM, pipetted through a 70 *μ*m cell strainer, plated on collagen type I-coated T-25 flask, and incubated for 2 hours (preplate 1; pp1). The media containing nonadherent cells were transferred into a second coated T-25 flask (pp2). After 18 hours, the medium from pp2 was centrifuged, the supernatant was removed, and the cell pellet was resuspended in the PM and transferred into a third T-25 flask (pp3). The previously described procedure was repeated until the pp6 culture was obtained. The last cell suspension was maintained for 72 hours. The cells were maintained in the PM at a low density (40–50% confluence) and expanded when needed. The medium was changed every 2-3 days. All the materials were purchased from Invitrogen (San Diego, CA, USA), unless defined differently.

### 2.4. Population Doubling Time

To evaluate the proliferative potential of BM-MSCs and MDSPCs, population doubling time (PDT) was determined. The cells were seeded in 6-well culture plates at a density of 9.5 × 10^4^ cells/well (10^4^ cells/cm^2^). The determination of the cell number was performed 6 times (passages 3–8) in 72 h intervals. Population doubling time assays were performed in triplicate for each isolated cell population. The PDT was calculated according to the following formulas [30]:

(1) PDT=CTPDNPDN=log⁡NN0×3.31,

where CT is time of cultivation between passages, PDN is population doubling number, *N* is cell number at the end of the cultivation period, and *N*0 is cell number at culture initiation.

### 2.5. Flow Cytometry Analysis

MDSPCs and BM-MSCs at passages 4-5 were analyzed by flow cytometry for mesenchymal stem cell markers CD90 and CD59, stemness marker c-kit (CD117), and hematopoietic and endothelial markers CD45 and CD34. All antibodies used for flow cytometry were purchased from Abcam, and CD59 was purchased from BD. The analysis was performed using fluorochrome-conjugated antibodies: CD34-phycoerythrin (PE), CD45-FITC, CD59-FITC, CD90-FITC, and CD117-biotin (c-kit). All incubations were conducted in the media composed of 0.5% BSA in PBS without calcium and magnesium. All antibody incubations were carried out for 30 min in the dark at room temperature and then washed with incubation media. Appropriately labelled isotype controls were used to define the specific gates. The analysis was performed on FACSCalibur with CellQuest software (BD Biosciences, San Diego, CA, USA).

### 2.6. Immunofluorescence Staining

MDSPCs and BM-MSCs at passages 4-5 were tested for CD34, CD45, CD90, c-kit, and desmin by immunofluorescence staining. All antibodies used for immunofluorescence staining were purchased from Abcam, and desmin was purchased from Sigma-Aldrich. Before staining, the cells were rinsed in PBS, fixed in 2% formaldehyde (Sigma-Aldrich; 10% formalin diluted in PBS) for 15 min, and rinsed again. Before antibody incubation, cells were blocked with either 10% horse serum or 10% goat serum for 1 hour, to permeabilise the cells and block nonspecific protein-protein interactions. Afterwards, the cells were incubated with primary antibody in PBS overnight at 4°C, followed by incubation with secondary antibody in PBS for 1 hour at room temperature, followed by incubation with streptavidin-Cy3 1 : 400 in PBS for 15 min (only for CD34, c-kit, and desmin), and counterstained with 4,6-diamidino-2-phenylindole dihydrochloride hydrate (DAPI; Sigma-Aldrich) 1 : 10,000 in PBS for 10 min. After each step, the cells were rinsed in PBS.

### 2.7. Gene Expression Analysis by RT-PCR

The expressions of stem cell genes POU class 5 homeobox 1 octamer-binding transcription factor 4 (OCT4), the NANOG homeobox (NANOG), and the sex determining region Y- (SRY-) box 2 (SOX2) of MDSPCs and BM-MSCs at passages 4-5 were tested using the semiquantitative real time polymerase chain reaction (RT-PCR). The total RNAs from the samples were extracted using ISOLATE II RNA Micro Kit (Bioline, UK) according to the manufacturer's instructions. Elution was performed with 10 *μ*L RNase-free water included in the kit. One-step RT-PCR was performed using Rotor-Gene Q 5-plex model (Qiagen, Germany). Rotor-Gene Q Series Software version 1.7 was used for the process. SensiFAST Probe No-ROX One-Step Kit (Bioline, UK), primers, and hydrolyzation probes (Biolegio BV, Netherlands) were used for one-step RT-PCR. 15 *μ*L of multiplex reaction contained 200 nM of each primer and 100 nM of each probe. The 2^−ΔΔCT^ method was applied for the relative gene expression data evaluation. *β*-actin gene expression was used for data normalization. RT-PCR reactions were carried out using the designed primers listed in Table 1.

### 2.8. Multipotent Differentiation

MDSPCs and BM-MSCs at passages 4-5 were tested for the adipogenic, chondrogenic, osteogenic, and myogenic differentiation potential* in vitro* using the previously described protocols [31]. Adipogenic, chondrogenic, and osteogenic media were purchased from Lonza.

#### 2.8.1. Adipogenesis

MDSPCs were seeded in collagen type I-coated 6-well plates and BM-MSCs in noncoated plates at a density of 2 × 10^5^ cells/well. On the second day, when the cells reached 100% confluence, cells were treated with 3 cycles of induction. One cycle consisted of 3 days in the adipogenic induction medium followed by 2 days in adipogenic maintenance medium. Thereafter, the cells were cultured in the adipogenic maintenance medium for 7 days. The control cells were cultured in the adipogenic maintenance medium for the entire period. Adipogenic differentiation was determined by oil red O staining.

#### 2.8.2. Chondrogenesis

The cells were aliquoted into 15 mL tubes at a density of 2.5 × 10^5^ cells/tube and centrifuged at 800 ×g for 5 min. The cells were then resuspended in chondrogenic basal medium, centrifuged again at 800 ×g for 5 min, resuspended in complete chondrogenic medium, and centrifuged at 500 ×g for 5 min. The complete chondrogenic medium was chondrogenic basal medium supplemented with 10 ng/mL transforming growth factor *β*3. The cell pellets were cultured in complete chondrogenic medium for 21 days, with medium change 3 times a week, embedded in the NEG50 freezing medium (Thermo Scientific, Kalamazoo, MI, USA), and snap frozen. The pellets were sectioned at 8 *μ*m thickness and fixed in 10% formalin (Sigma-Aldrich) for 10 min. Chondrogenic differentiation was confirmed by Alcian blue staining.

#### 2.8.3. Osteogenesis

Similarly, as for chondrogenesis, cells were aliquoted into 15 mL tubes at a density of 2.5 × 10^5^ cells/tube, centrifuged, resuspended in the osteogenic medium, and centrifuged at 500 ×g for 5 min. The cell pellets were cultured for a total of 4 weeks, with medium change 3 times a week. Afterwards, the pellets were embedded in the NEG50 freezing medium and snap frozen. The pellets were then cut into 8 *μ*m sections and fixed in 10% formalin for 10 min. Mineralization was confirmed by von Kossa staining.

#### 2.8.4. Myogenesis

MDSPCs were seeded in collagen type I-coated 12-well plates and BM-MSCs in noncoated plates at a density of 6 × 10^4^. On the second day, at 100% confluence, the cells were shifted to the myogenic medium, which consisted of high-glucose DMEM, supplemented with 2% FBS and 1% P/S. The medium was changed 3 times a week for 2 weeks. Myogenic differentiation was determined using desmin immunofluorescence.

### 2.9. Nephrotoxicity Model

AKI was induced by intraperitoneal injection of gentamicin at 80 mg/kg daily for 7 consecutive days. The model design (nephrotoxicant; its dosage and monitoring time) was based on our previous experiments [32]. Rats were randomly divided into 3 groups (*n* = 6 for each group per time point): Control group (healthy rats), GM group (gentamicin injections only), and GM + MDSPCs group (gentamicin injections plus MDSPCs injection). A single MDSPCS injection (1 × 10^6^ cells/500 *μ*L serum-free medium) was administered intravenously into the tail vein 24 hours after the last gentamicin injection. Blood, urine, and tissue samples were collected for the determination of the renal function and tissue damage. Blood and urine samples were analyzed using automatic biochemistry analyzer COBAS INTEGRA 400 plus (Tegimenta Ltd. Roche, Switzerland). Urine and blood samples were collected on day 0 (24 hours before gentamicin injection), day 8 (24 hours after the last gentamicin injection), day 14, and day 21. Urine volume per 24 hours (*V*_U/24 h_) and urinary (*U*_Cr_) and serum (*S*_Cr_) creatinine levels were determined. Glomerular filtration rate (GFR) was calculated according to the creatinine clearance (*C*_Cr_) as follows:

(2) $$\begin{array}{c} C_{Cr}=\frac{\left(U_{Cr}\times V_{U/24\;h}\right)}{\left(S_{Cr}\times 24\times 60\right)}. \end{array}$$

**Figure 1:**
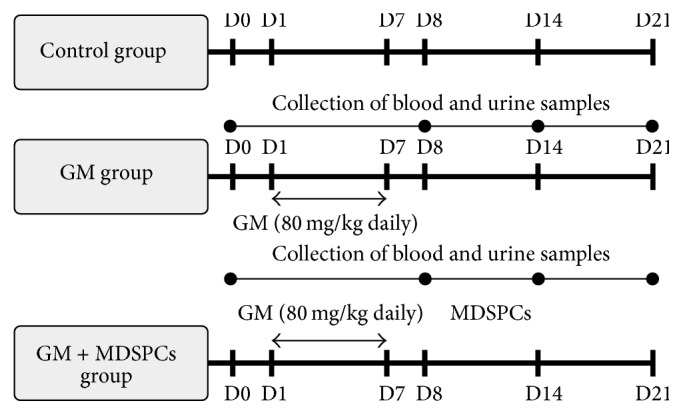
Experimental study design flowchart. AKI was induced by intraperitoneal injection of gentamicin at 80 mg/kg daily for 7 consecutive days. Rats were randomly divided into 3 groups (*n* = 6 for each group per time point): Control group (healthy animals), GM group (gentamicin injections only), and GM + MDSPCs group (gentamicin injections plus MDSPCs injection). A single MDSPCS injection (1 × 10^6^ cells/500 *μ*L serum-free medium) was administered intravenously into the tail vein 24 hours after the last gentamicin injection (D8). Blood, urine, and tissue samples were collected to evaluate renal function and tissue damage. Urine volume, urinary (*U*_Cr_) and serum (*S*_Cr_) creatinine levels, and glomerular filtration rate (GFR) were determined at day 0 (24 hours prior to gentamicin injection), day 8, day 14, and day 21 of the experiment.

The rats were euthanized on day 8 in order to validate the nephrotoxicity model and later on day 14 and day 21 of the experiment. The schematic representation of an* in vivo* experimental model setup is shown in Figure 1.

### 2.10. Renal Histology

Kidney specimens from all the animals were fixed in 10% buffered formalin before embedding in paraffin. The tissue was sectioned at 5 *μ*m and then stained with haematoxylin and eosin (HE) and periodic acid–Schiff (PAS) for light-microscopy analysis. The histological analysis was processed by 3 independent researchers in a blind fashion. Tubular necrosis, loss of brush border, cast formation, and tubular dilatation were evaluated in 10 randomly chosen, nonoverlapping fields of each section as follows: 0, 0%; 1, ≤10%; 2, 11–25%; 3, 26–45%; 4, 46–75%; and 5, 76–100%. Tubular injury was scored by calculating the percentage of the affected tubules. The whole tubule numbers per field were considered as standard. The percentage of tubular injury was calculated in each field as follows:

(3) Renal  injury  score  %=Number  of  injured  tubulesNumber  of  whole  tubules×100.

### 2.11. *In Vivo* Tracking of Muscle-Derived Stem/Progenitor Cells

For* in vivo* tracking, MDSPCs were labelled with the red fluorescent membrane dye PKH-26 according to the instructions provided by the manufacturer (Sigma-Aldrich) just prior to the injection. The rats were sacrificed 7 and 14 days after MDSPCs administration (on days 14 and 21 of the experiment); the kidney samples were snap frozen and kept at −80°C until examination. The frozen samples were sectioned at 5 *μ*m; the nuclei were stained with DAPI. Fluorescent microscopy was performed with Olympus IX73 microscope using QCapture Pro 7 image and analysis software.

### 2.12. Statistical Analysis

Statistical analyses were performed using the SPSS Statistics 17.0 software package. All quantitative data are expressed as the mean ± standard deviation. Student's *t*-test was used for comparison between the 2 groups. Analysis of variance (ANOVA) and Bonferroni* post hoc* test were used for multigroup comparison. A value of *p* < 0.05 was considered statistically significant.

## 3. Results

### 3.1. Isolation and Culture of MDSPCs and BM-MSCs

**Figure 2:**
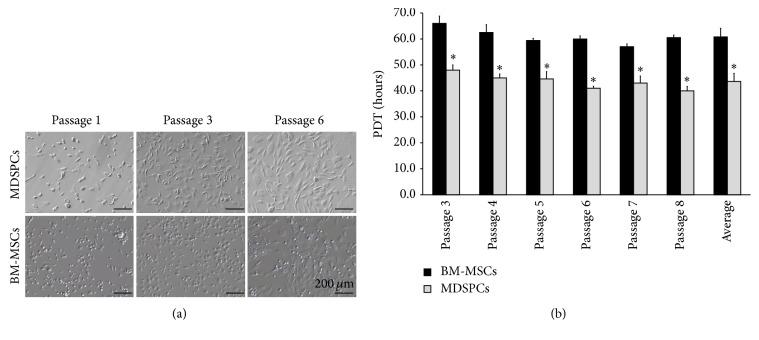
Morphology and proliferation analysis of MDSPCs and BM-MSCs. (a) The BM-MSCs during the first day of culture appeared as round-shaped cells. After 3-4 days, the adherent cells became spindle-shaped (BM-MSCs, passage 1). BM-MSCs were passaged after reaching 65–70% confluence (BM-MSCs, passages 3, 6). MDSPCs appeared as round, triangular, or spindle-shaped (MDSPCs, passage 1). MDSPCs were passaged after reaching 65–70% confluence (MDSPCs, passages 3, 6). (b) MDSPCs had significantly higher proliferation rate (*p* < 0.05) in comparison to BM-MSCs, as reflected by the average population doubling time. The average PDT of MDSPCs was 43.64 ± 3.10 hours, while BM-MSCs exhibited a PDT of 60.78 ± 3.34 hours. ^*∗*^ *p* < 0.05, significant difference.

We successfully isolated mesenchymal stem cells from the rat bone marrow and the skeletal muscle. The isolated BM-MSCs during the first day of culture adhered to plastic culture flask and appeared as round-shaped cells. After 3-4 days, the initial adherent cells became more spindle-shaped (Figure 2(a), BM-MSCs, passage 1). Within 5–10 days, the cells began to proliferate more rapidly and reached 65–70% confluence within 14 days. MDSPCs were isolated using the preplate technique. The pp6 culture was considered to be passage 1. Most of the nonadherent cells seeded in the pp6 died, but the surviving adherent cells slowly began to proliferate. These viable cells appeared as small, round, triangular, or spindle-shaped (Figure 2(a), MDSPCs, passage 1). Within 6-7 days, the cells reached 65–70% confluence.

### 3.2. Population Doubling Time

MDSPCs tended to double the population in the average of 43.64 ± 3.10 hours while BM-MSCs exhibited a PDT of 60.78 ± 3.34 hours, and this ~17-hour difference was statistically significant (*p* = 0.001). The PDT of BM-MSCs and MDSPCs of passages 3 to 8 is shown in Figure 2(b).

### 3.3. Phenotypic Characterization and Gene Expression Patterns

**Figure 3:**
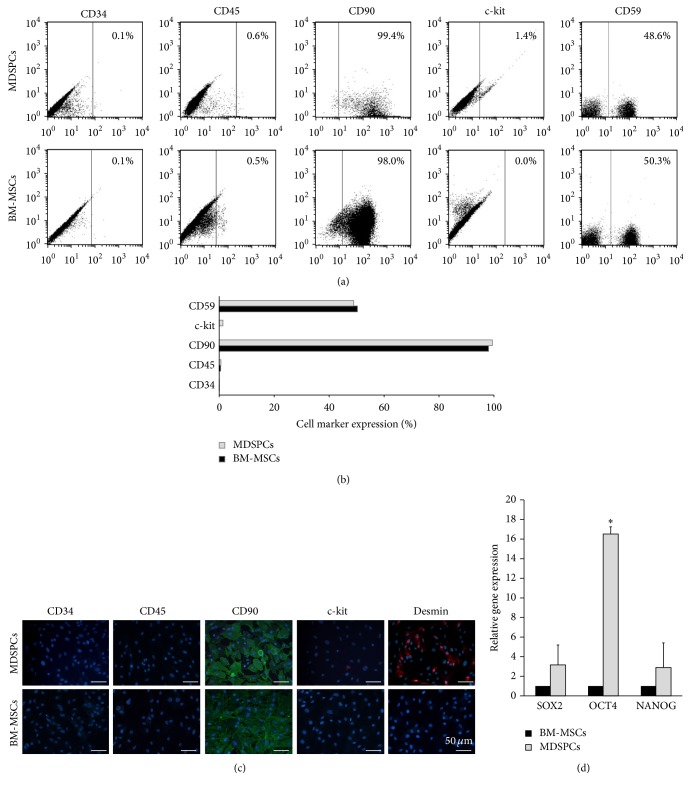
Surface marker and gene expression analysis of MDSPCs and BM-MSCs at passages 4-5. Surface marker expression was determined by the flow cytometry ((a), (b)) and immunofluorescence staining (c). BM-MSCs and MDSPCs highly expressed CD90 and CD59 and were negative (<1%) for CD45 and CD34. MDSPCs were positive for desmin and weakly positive for c-kit (CD117), while BM-MSCs were negative for both of these markers. (d) MDSPCs had higher expression of SOX2, OCT4, and NANOG, but the significant difference was detected only in OCT4 expression (*p* = 0.037). ^*∗*^ *p* < 0.05, significant difference.

BM-MSCs and MDSPCs revealed quite similar expression patterns of surface markers as determined by the flow cytometry and immunofluorescence staining (Figures 3(a)–3(c)). BM-MSCs and MDSPCs highly expressed mesenchymal stem cell markers thy-1 CD90 and MAC-inhibitory protein CD59 and were found negative (<1%) for hematopoietic and endothelial markers CD45 and CD34. MDSPCs were also found to be positive for muscle-specific type III intermediate filament desmin, showing their origin from the skeletal muscle, and weakly expressed stem cell growth factor receptor c-kit (CD117), while BM-MSCs were found to be negative for both of these markers.

The quantitative RT-PCR analysis revealed a consistent stemness marker expression profile in all the MDSPCs populations examined which was compared to the gene expression of BM-MSCs. The RT-PCR results demonstrated that MDSPCs had a significantly higher expression of OCT4 (*p* = 0.037), while NANOG and SOX2 expression was similar in both cell lineages (Figure 3(d)).

### 3.4. Multilineage Differentiation Capacities* In Vitro*

**Figure 4:**
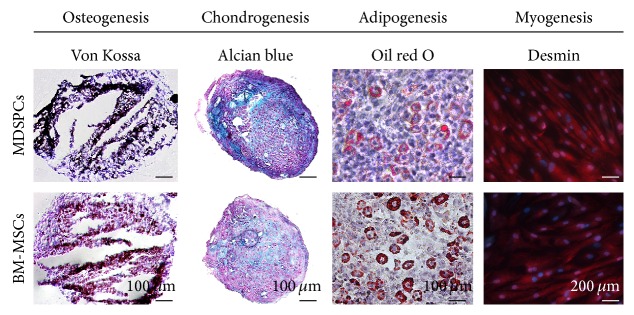
Multilineage differentiation of MDSPCs and BM-MSCs. Cells cultured in osteogenic differentiation medium formed bone nodules as determined by von Kossa staining. Cells cultured in chondrogenic differentiation medium had lacunae surrounded by the Alcian blue-stained matrix. Cells cultured in adipogenic medium formed lipid droplets, which were positive for oil red O staining. BM-MSCs and MDSPCs differentiated into myogenic lineage and became multinucleated, elongated, and highly positive for desmin (>98%).

Both BM-MSCs and MDSPCs were capable of multilineage differentiation (Figure 4). The cells which followed 3 cycles of adipogenesis induction during a period of 3 weeks had positive results for oil red O staining of lipid droplets. The cells cultured in the standard medium did not develop lipid droplets and lacked oil red O staining. BM-MSCs and MDSPCs cultured in chondrogenic differentiation medium for 21 days had cells within lacunae in Alcian blue-stained matrix. Cells cultured in standard medium did not have lacunae and lacked Alcian blue-positive matrix. The cells cultured in the osteogenic differentiation medium for 4 weeks formed bone nodules as determined by von Kossa staining. The cells cultured in the standard medium did not develop nodules and lacked von Kossa staining. After 2 weeks of induction, BM-MSCs and MDSPCs demonstrated the ability to differentiate into the myogenic lineage. At the end of the experiment, the cells became multinucleated and elongated and were highly positive for desmin (>98%).

### 3.5. Effects of MDSPCs on Kidney Function after AKI

**Figure 5:**
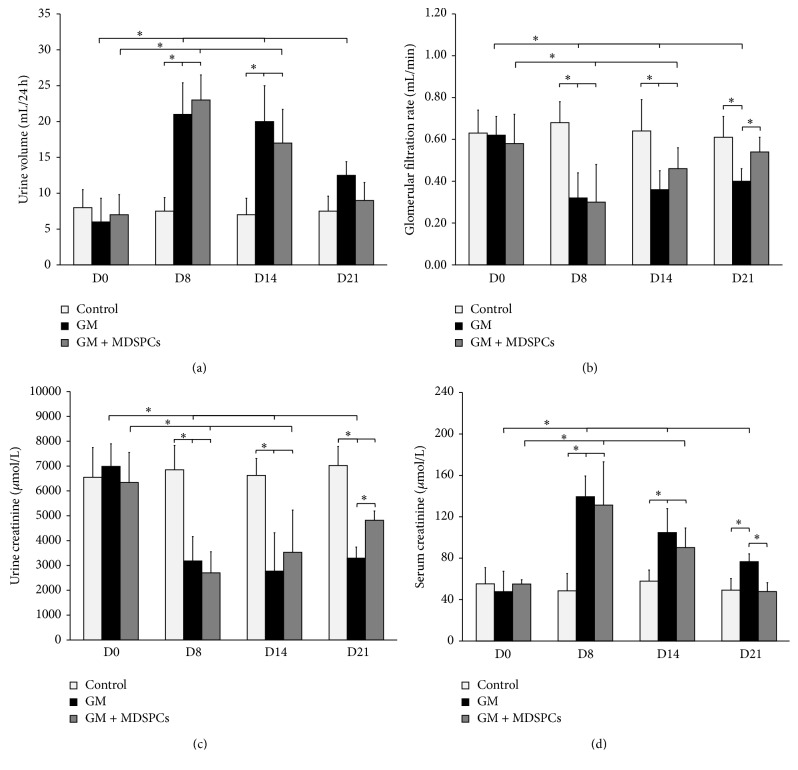
MDSPCs impact on functional recovery of damaged kidney. Gentamicin-induced acute kidney injury was validated by the presence of physiological and functional changes in all rats from GM and GM + MDSPCs groups ((a), (b), (c), and (d), D8). There was no significant difference in urine volume between GM and GM + MDSPCs groups at any time point ((a); *p* > 0.05). The MDSPCs accelerated the recovery after AKI, as reflected by significantly higher GFR ((b); *p* = 0.034), increased urinary creatinine levels ((c); *p* = 0.015), and lower serum creatinine ((d); *p* = 0.030) as compared to the GM group rats that were not treated with MDSPCs. ^*∗*^ *p* < 0.05, significant difference.

Four time points (days 0, 8, 14, and 21) were chosen for the determination of the serial changes in rat urine volume, serum, and urinary levels of creatinine and GFR. There was no significant difference in any parameters on days 0, 8, and 14 between GM and GM + MDSPCs. There was no significant difference in any parameters on days 0, 8, 14, and 21 in Control group animals. Similarly, no significant differences were found between all 3 groups on day 0. The physiological and functional changes were present in rats from the GM and GM + MDSPCs groups on days 8 and 14, demonstrating gentamicin-induced acute kidney injury, and these differences were statistically significant in comparison with parameters on day 0 (*p* < 0.05) and in comparison with Control group on days 8 and 14 (*p* < 0.05). As shown in Figure 5, MDSPCs accelerated the recovery after AKI, as reflected by significantly lower serum creatinine (*p* = 0.030), increased urinary creatinine levels (*p* = 0.015), and GFR (*p* = 0.034) compared with the GM group rats that were not treated with MDSPCs. There was no significant difference in the average urine volume between the groups on day 21.

### 3.6. Effects of MDSPCs on Kidney Histology after AKI

**Figure 6:**
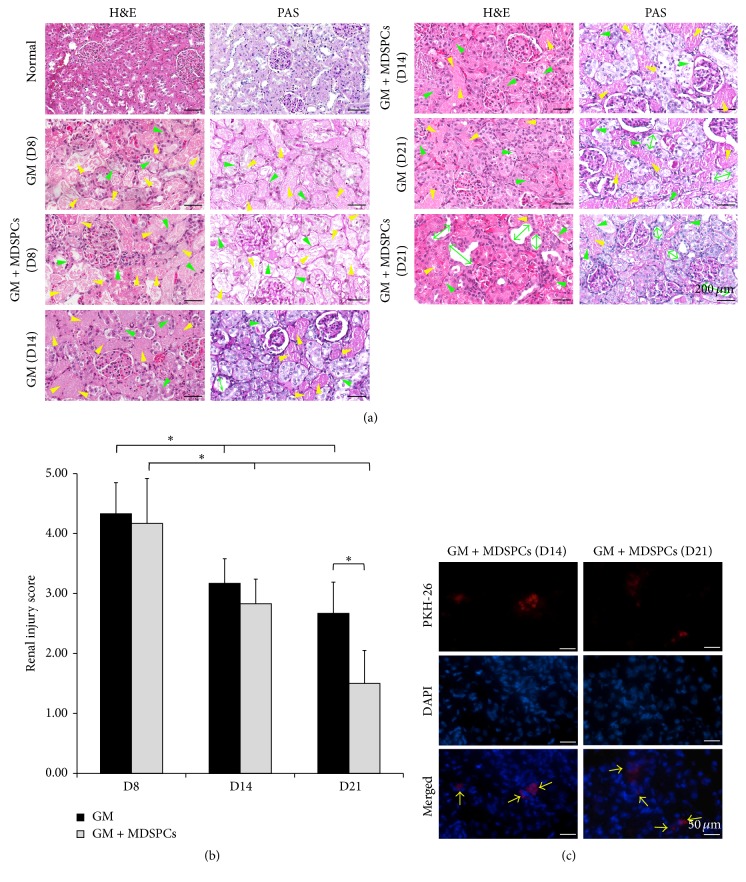
MDSPCs impact on morphological recovery after AKI and their presence in the damaged renal tissue during the period of 2 weeks. Gentamicin injections at 80 mg/kg for 7 consecutive days caused typical aminoglycoside-induced AKI, including the tubular necrosis (yellow arrow heads), loss of brush border in renal tubules (green arrow heads), and tubular dilatation (green both-way arrows) ((a); GM (D8) and GM + MDSPCs (D8)), represented by the renal injury score of 4.33 ± 0.52 and 4.17 ± 0.75, respectively (b). MDSPCs injection attenuated renal tubular damage ((a); GM + MDSPCs (D21)) and significantly lowered renal injury score to 1.5 ± 0.55, compared with the injury score of 2.67 ± 0.52 in GM group after 21 days ((b); *p* = 0.008). PKH-26-labeled MDSPCs were detected within the renal cortex, localized primarily in the renal tubules and the interstitial compartment of the kidney 7 and 14 days after the MDSPCs administration ((c); yellow arrows). ^*∗*^ *p* < 0.05, significant difference.

Gentamicin injections at 80 mg/kg for 7 consecutive days caused typical aminoglycoside-induced AKI, including the tubular necrosis, cast formation, loss of brush border in the renal tubules, and tubular dilatation (renal injury score of 4.33 ± 0.52 in GM group and 4.17 ± 0.75 in GM + MDSPCs group). Even though 7 days after the last gentamicin injection kidney injury was considerably decreased in GM + MDSPCs group in comparison to GM group (renal injury 3.17 ± 0.41% versus 2.83 ± 0.41%), this difference was not statistically significant. MDSPCs injection significantly attenuated renal tubular damage, as shown by the kidney histology (Figure 6(a)) and significantly lower renal injury (Figure 6(b)) score of 1.5 ± 0.55 (*p* = 0.008) in the GM + MDSPCs group, compared with the injury score of 2.67 ± 0.52 in the GM group after 21 days of the experiment (2 weeks after the injection of MDSPCs).

### 3.7. *In Vivo* Tracking of MDSPCs

The existence of MDSPCs in the renal tissue was evaluated by the presence of PKH-26-labeled cells in the kidney sections, 7 and 14 days after the administration of MDSPCs (on day 14 and day 21). PKH-26-labeled MDSPCs were identified within the renal cortex and localized primarily in the renal tubules and the interstitial compartment of the kidney (Figure 6(c)). No PKH-26 positive cells were detected in the renal tissue of GM group on day 14 and day 21 (data not shown).

## 4. Discussion

The results of the present study indicate that rat MDSPCs and BM-MSCs have very similar immunophenotype, gene expression, and multilineage differentiation potential. However, the MDSPCs exhibited higher proliferation capacity. The PDT of MDSPCs appeared to be significantly lower than the PDT of the BM-MSCs, demonstrating the advantage of MDSPCs having a more rapid proliferation rate. The results of our experiments* in vivo* have shown that a single injection of MDSPCs accelerated the functional recovery and significantly enhanced kidney regeneration after gentamicin-induced renal damage. In addition, MDSPCs have the capacity of migrating into the injured renal tissue and populating the renal cortex.

For the last few decades, researchers have been eagerly looking for the novel therapies in AKI setting, including advanced therapies, that is, various stem cell preparations or their components, primarily using bone marrow mesenchymal stem cells [14–20, 33–36]. Currently, there is no clear understanding on the mechanism of action of cellular therapies. The proposed mechanisms are focused on several factors, implanted stem cells acting directly or through paracrine/endocrine effects on renal progenitor cells. Thus, we aimed to compare the mesenchymal stem cells derived from the skeletal muscle and the bone marrow. Our results regarding the characterization of both MDSPCs and BM-MSCs are similar to those of other studies. Both cell types isolated from rats, horses, and humans were found to be highly positive for CD90 and negative for CD34 and CD45 [37–39]. MDSPCs were also positive for desmin, showing their origin from the muscle tissue. Both cell types were capable of adipogenic, chondrogenic, osteogenic, and myogenic differentiation* in vitro* [37–39]. The most important difference between BM-MSCs and MDSPCs determined in our study was the growth kinetics. MDSPCs were found to have significantly lower PDT in comparison to BM-MSCs, similar to the previously reported data [37]. These results indicate the advantage of MDSPCs, as the required cell number for therapeutic purposes can be obtained in a shorter period of time. Moreover, considering the possible future application of MDSPCs in the clinical practice, muscle tissue biopsy would be less invasive, less painful, and, thus, better tolerated by the patients.

Tubular necrosis, cast formation, loss of brush border in the renal tubules, and tubular dilatation are characteristic features of gentamicin-induced nephrotoxicity, which results in kidney dysfunction. Functional and histological renal changes induced by gentamicin have been reported* in vivo* [40–42]. Using the rat model, we have shown that a single intravenous injection of MDSPCs after gentamicin-induced AKI significantly improved the renal function and altered renal remodelling in comparison with gentamicin-injured animals not given MDSPCs. The improvement of renal function was associated with the reduced serum creatinine level and the increased urinary creatinine level and GFR. In addition, MDSPCs administration also reduced the renal injury score, which is an important feature of the renal failure. The effect was very similar to that reported with the mesenchymal stem cells derived from the bone marrow. The BM-MSCs were able to minimize the renal damage in different models of AKI, such as the gentamicin, cisplatin, or ischemia/reperfusion experimental models [14–16, 18–20, 34–36].

One of the biggest challenges with respect to cell therapy in the kidney is the understanding of the mechanisms involved in the therapeutic effect. The possible cellular factors that initiate the recovery phase in kidney regeneration after the treatment with stem cells remain controversial. Among the possible mechanisms of action of BM-MSCs for the treatment of AKI are the reduction of cell apoptosis and the anti-inflammatory effects [14, 16, 33]. Similar results were presented in other tissue injury models, demonstrating the antiapoptotic and anti-inflammatory effects of MDSPCs [27, 43, 44]. In our study, we aimed to assess whether there was any targeted kinetics of MDSPCs to the damaged tissue and demonstrated the presence of MDSPCs in the renal tissue up to 2 weeks after administration. This supports the previous findings that stem cells improve the kidney function and structure directly, by migrating to the kidney and populating the renal cortex as suggested by other authors [17–19, 34]. Considering the fact that in our study the significant improvement of the kidney function and histology was observed even though the amount of MDSPCs in the renal tissue was relatively low, we cannot exclude the possible paracrine/endocrine effect reported by other researchers as the main mechanism for systemically injected stem cells [14–16, 20, 33, 36]. Further research is needed to address this issue.

To date, this is the first study investigating MDSPCs isolated using preplate isolation technique [12], as a potential new treatment for gentamicin-induced AKI. Although Arriero et al. have reported that one type of stem cells derived from skeletal muscle has no beneficial effect for the treatment of AKI [45], they acknowledged the clear differences between the isolation methods and phenotypes of the stem cells isolated by authors' group in comparison with the preplate isolation technique of MDSPCs. The authors have demonstrated that stem cell population isolated from skeletal muscle by their group was c-kit negative and displayed rare expression of hematopoietic stem cell marker CD34, while MDSPCs isolated by our group were found negative (<1%) for CD34 and weakly expressed stem cell growth factor receptor c-kit. In addition, Arriero et al. have investigated the effect of skeletal muscle-derived stem cells for renal dysfunction after acute ischemia, while in our study we have used gentamicin-induced AKI model. In our study, the stem cell dose used for the treatment was 5 times higher than that in the previously reported study (1 × 10^6^ versus 2 × 10^5^ cells/animal). The authors reported that the transplantation of undifferentiated muscle-derived stem cells had no effect on the renal function recovery, while our study revealed the considerable effect of MDSPCs therapy in accelerating recovery and regeneration of the damaged kidney. This reiterates that the skeletal muscle might be a source of several types of stem cells [23] and the MDSPCs isolated using the preplate isolation technique might possess the capacity to regenerate the injured renal tissue.

Another strength of this study includes the comprehensive* in vitro* comparison of MDSPCs and BM-MSCs isolated from rat. Even though there are previous data that demonstrate similarities and differences of these cell lineages, such comparisons were made using cells derived from other species, including murine, lapine, canine, equine, or human [38, 46–50].

We demonstrated the feasibility of MDSPCs application and the proof of the concept for the treatment of AKI. Besides the novelty of our study, several questions remain unanswered. The main mechanism of action of MDSPCs remains questionable. The possible antiapoptotic and anti-inflammatory effects of MDSPCs should be researched in the AKI models, as these mechanisms were previously reported using BM-MSCs [14, 16, 33]. Further research is necessary to determine the differences between the therapeutic effect of BM-MSCs and MDSPCs for the treatment of AKI, as well as the most appropriate route of administration (intravenous versus intraparenchymal injection) and the optimal dosage of cells. It is also important to assess whether the therapeutic effect can be enhanced by repeated injections of MDSPCs, as well as possible long-term outcomes (4–8 weeks) after the treatment. Combination therapy with substances such as antioxidants may also be one of the directions for further research, as previous studies have reported superior results using BM-MSCs with other agents for the treatment of AKI in comparison with BM-MSCs therapy alone [51, 52].

In summary, we have demonstrated that the rat MDSPCs represent an alternative source of mesenchymal stem cells possessing several advantages over those derived from bone marrow, including abundance of skeletal muscle tissue, relatively easy cell isolation and expansion technique, and most importantly higher proliferation rate. We also show that MDSPCs have the beneficial impact on functional and morphological recovery of damaged kidney. The cellular therapy with MDSPCs may become a potential new strategy for the treatment of AKI in the experimental studies and must be further investigated.

## Figures and Tables

**Table 1:** Primer sequences and conditions for real time reverse transcription polymerase chain reaction.

Gene	Primer nucleotide sequence	Product size (bp)	Annealing temperature (°C)
*β*-actin	5′-GCACMATGAAGATCAAGATCATTGCTCC-3′ (forward)	118	60
	5′-TCRTACTCCTGCTTGCTGATCCAC-3′ (reverse)		

Oct4	5′-GGCCCCTGCTGGAGAAGTG-3′ (forward)	120	60
	5′-CACGGTTCTCAATGCTAGTCCGC-3′ (reverse)		

Sox2	5′-TCAGCGCCCTGCAGTACAAC-3′ (forward)	140	60
	5′-GGCCTCGGACTTGACCACAG-3′ (reverse)		

NANOG	5′-GGTTGAAGACTAGCAACGGTCTGACT-3′ (forward)	81	60
	5′-AGCCCTGAGAATAGCTGCAATGG-3′ (reverse)		

## Abbreviations

AKI: Acute kidney injury
BM-MSCs: Bone marrow mesenchymal stem cells
BSA: Bovine serum albumin
DAPI: 4,6-Diamidino-2-phenylindole dihydrochloride hydrate
DMEM: Dulbecco's modified eagle medium
FBS: Fetal bovine serum
FITC: Fluorescein isothiocyanate
GFR: Glomerular filtration rate
GM: Gentamicin
HBSS: Hank's balanced salt solution
HE: Haematoxylin and eosin
MDSPCs: Muscle-derived stem/progenitor cells
MSCs: Mesenchymal stem cells
OCT4: Octamer-binding transcription factor 4
P/S: Penicillin-streptomycin
PAS: Periodic acid–Schiff
PBS: Phosphate buffered saline
PDT: Population doubling time
PE: Phycoerythrin
PM: Proliferation medium
RT-PCR: Real time polymerase chain reaction
SOX2: Sex determining region Y- (SRY-) box 2.
